# Curriculum and the Covid-19 crisis

**DOI:** 10.1007/s11125-021-09560-y

**Published:** 2021-06-21

**Authors:** William F. Pinar

**Affiliations:** Department of Curriculum and Pedagogy, Vancouver Campus, University of British Columbia, 2125 Main Mall, Vancouver, BC V6T 1Z4 Canada

**Keywords:** Curriculum, Covid-19, Crisis, Technology

## Abstract

The article argues that the Covid-19 crisis is a curriculum crisis, because it is a humanitarian crisis. Survival—physical, psychological, educational—is at stake. As educators have mobilized to meet the emergency, this introductory article to *Prospects* special issue on *Curriculum Responsiveness to Crisis* glimpses elements of that effort, both theoretical and practical. It concludes that the student—the individual person—should remain central to any conception of curriculum, to any organization of pedagogical communication, indeed to the very project of education itself.

The curriculum is often organized around the school subjects, themselves modeled after academic disciplines at the university, an arrangement sealed by standardized assessments, a pattern interrupted during the Covid-19 pandemic. Schools shuttered, instruction moved online, the curriculum cracked. First and foremost a public health crisis, the pandemic is also an economic and educational crisis, forcing many to struggle while sheltering in place, social distancing, attempting to teach and learn with technologies teachers and students frequently found frustrating. While in wealthy countries many complained about online learning, in poorer places hundreds of millions of children lacked that privilege, as they had no computers or access to the internet. These children lost access to schooling altogether. And not only in poorer places: New York City failed to provide internet access to some 111,000 children living in homeless shelters and unstable housing; for these children online learning was no option either (Gettleman and Raj 2020). Unemployed and economically desperate parents pressed their children into labor; there were reports of children mining sand in Kenya; laboring on cocoa plantations in West Africa; and painted silver, posing as living statues begging for money in Indonesia (Gettleman and Raj 2020). This surge in child labor erased recent gains in school enrollment, literacy, social mobility, and children’s health: “All the gains that have been made, all this work we have been doing, will be rolled back, especially in places like India”, lamented Cornelius Williams, a UNICEF official” (A1). “Child labor is just one piece of a looming global disaster”, Gettleman and Raj reported, as “severe hunger is stalking children from Afghanistan to South Sudan” (A1). United Nations officials reported a rise in forced marriages for girls and child trafficking generally, especially across Africa and Asia. Teen pregnancies in Uganda increased during pandemic-related school closures; in Kenya, “many families” forced their teenage girls into sex work to feed the family (Gettleman and Raj 2020). All the while, the pandemic and the inequitable distribution of vaccines threatened, maybe “most of all”, 70 million refugees, “the least likely to have soap and water, food and medicine” (Dorfman 2020, p. 50).

The Covid-19 crisis is a curriculum crisis because it is a humanitarian crisis. Survival—physical, psychological, educational—is at stake. Educators mobilized to meet the emergency. We glimpse elements of that effort, both theoretical and practical, in this special and enlarged issue of *Prospects*. You will find articles focused on this “new normal” (Pacheco), what that might mean for the post-Covid-19 curriculum (Aktan), in this era of “uncertainty” (Goodson and Schostak). While some were “tinkering” with the curriculum (Amin and Mahabeer), others were confronting what the pandemic meant for our “consciousness” (Poindexter, Smith, and Wang), the pandemic as another iteration of “the plague” (Murillo), affecting our sense of “time” (Spector), as time itself seemed suspended and “nothing happened” (Block). You will find an article on early childhood (Spiteri), on the “prophetic” (Burns and Cruz), “flipping” (Backes et al.), “re-imagining” (Courtland, also Batra), as well as understanding, the curriculum as “geo/biospheric text” (Bigloo, Scott, and Adler). There is an article on “embodiment” (Kasamali), on “anti-science” (Lopes), on “Indigenous resilience” (Brant-Birioukov), “global citizenship” (Kim), “planetary responsibility” (Ranniery), “decolonizing nature” (Foley), all the while “dreaming” (Neufeld) of a “thoughtful lightness” (Phelan and Hansen), each of these efforts provoked by this long and momentous “event” (Morelli) that forced almost everyone into “teaching remotely” (Strong-Wilson and Yoder), a “viralization of education” (Rossini, Amaral, and Santos), providing “opportunities for new governance” (Macedo), engendering a “period of transition” (Durygin), an era of “liminality, disruption, and change” (Fisher et al.), requiring a “curriculum response” (Charland et al.), a “rethinking of standardized senior secondary examinations” (Cairns), emphasizing “relevance” (Ramrathan), perhaps a curriculum of embodiment (Kasamali), while we consider its “prospects” (Le Grange), including “community participation” (Iyengar), “clinical practice” (Hadar, Alpert, and Ariav), “hygiene and disease prevention” (Morrish and Neesam), “biology curricula of the future” (Roberts), each of a form of “curricular responsiveness” (Aletheiani; see also Gul and Khilji), if not a rethinking of “basic education” (Hakala and Kujala).

I dwell on the pandemic’s intensification of the technologization of education, a phenomenon well underway before the Covid-19 crisis but accelerated by it. For over a century, profiteers have promised solutions to educational problems if only we use the devices they produce. Those devices have certainly shifted over time, but the hype never ends. Forced online, staring at screens, many children and their parents first felt frustrated, then disillusioned, as the reports I cite here confirm. This realization that technology cannot substitute for actual teachers and earnest academic study will, I suspect, change nothing except the products produced. That conclusion is foreshadowed in the abbreviated history I will sketch—starting with slides in the late 19th century, followed by enthusiasm for radio, television, teaching machines, and computers in the 20th-technologists respond to public disillusion by offering something “new”, prompted by profits and by promoting the faith that technology can solve any problem, even the problems technology itself creates. While not denying humanity’s dependence on, indeed our embeddedness in, technology, my admittedly impressionistic review—drawing upon journalists’ reports—confirms that once again our experience of educational technology did not align with our confidence in it. Technology, however, was not the only issue in play during the crisis.

## Suffering, struggle, survival

While the pandemic pressed professional educators to rethink, restructure, and reimagine what curriculum is and can be, it pressed others into criminality. With Nigeria’s economy in crisis, kidnapping increased; its victims were not only the rich, powerful, or famous but also the poor and, increasingly, schoolchildren, sometimes seized *en masse*. The perpetrators were gangs of bandits, exploiting inadequate policing and easy access to guns (Maclean 2021). “Each kidnapping seems to inspire another”, Maclean reported, and the “media coverage that erupts after every incident puts pressure on the government to win the release of the hostages” (A10). Often located in Nigeria’s northwest, sometimes far from the security cities and towns provide, boarding schools have been especially vulnerable.

Not only in Nigeria did the public health crisis bring economic crisis and the moral corruption it encourages. In the United States, US Secretary of Education Betsy DeVos allowed the Paycheck Protection Program (PPP; part of the 2020 CARES Act, legislation designed to rescue small business from bankruptcy) to be exploited by private, religious, and charter (i.e., schools that receive government funding but operate independently of the established state school system) schools. Private and religious schools received an average of about $855,000 each, compared with about $134,500 per public school; religious schools, elite private schools, and more than one thousand charter schools took from $150,000 to $10 million each. The one-thousand-student Buckingham Browne and Nicholas elite private school in Cambridge, Massachusetts, a school that collects $52,300 in tuition and is supported by an endowment of $75 million, received between $5 million and $10 million (Ravitch 2021). “The Paycheck Protection Program”, Ravitch reported, “turned out to be a multibillion-dollar bonanza for non-public and religious schools, at a time when most public schools lacked the funding to pay for social distancing, health measures, and personal protection equipment for students and staff” (p. 36).

Corruption knows no ideology, but right-wing administrations—such as that of former US President Donald Trump—seem especially hospitable to it, including non-economic and specifically intellectual forms; for instance, pressuring scholars to rescind findings unwelcome by right-wing rulers. A case in point occurred in Poland, where a judge ruled that two Holocaust scholars must issue a public apology for including what was alleged to be “inaccurate information” in a two-volume academic study documenting the role played by individual Poles in the murder of Jews during World War II (Higgins 2021). The ruling was made at the end of a closely watched libel trial brought by the niece of a wartime village mayor who, according to a Jewish survivor cited in a 2018 study coedited by the two scholars, had been complicit in the murder of 18 Jews who took shelter from the Nazis in a forest in eastern Poland. The two scholars are Jan Grabowski, a Polish-Canadian history professor at the University of Ottawa, and Barbara Engelking, a historian at the Polish Center for Holocaust Research, which had published the book in 2018. Titled *Night Without End*, the book is a 1700-page study of Polish conduct under Nazi occupation from 1939 to 1945, during which time, some three million Jews were murdered within Polish borders, most in Nazi death camps but also sometimes by Poles (Higgins 2021).

In the United States, events in early January 2020 posed problems for educators across the country: the right-wing riot at the US Capitol, the second impeachment of then-President Trump, and the transfer of power from Trump to newly elected Joseph Biden were all politically charged topics (Nierenberg 2021). “Across the United States”, Nierenberg reported, “educators have rerouted their syllabuses towards the news”, whether teaching science fiction, Shakespeare, or the history of ancient Rome (A1). Whatever their subject, educators looked for “parallels to help their students process the often frightening and surely historic events” (A18); I theorize such “parallels” as “allegories of the present” (Pinar 2019, p. 27). In the politically polarized United States, even observing common-sense public health measures—wearing masks, the safe reopening of schools—became controversial.

After starting classes remotely in September 2020, the Wausau (a city of 38,000 in central Wisconsin) school board capitulated to community pressure and reopened schools in November, the very month the pandemic was “surging” across the state (Bosman 2021, A20). Hundreds of students were exposed to the virus. There were no staff or student deaths, but several staff members were hospitalized. School board members “turned on one another in a bitter quarrel that frustrated parents, making it and the administration a lightning rod in the community” (A20). Similar conflicts occurred across the United States—by then, the global center of the pandemic—as school board members were pressured to become “instant public health experts, balancing teacher concerns about safety with the educational needs of students and the burdens on working parents” (A20). “The teachers have really been trying, going out of their way to communicate, but there’s been virtually no human contact for months and months”, one parent (Julie Regalado, whose daughter is a high school freshman) told reporter Jennifer Medina (2021): “[learning] virtually is nobody’s dream. But I cannot imagine my daughter going back at all this year, since we’re seeing a rise in cases every day” (pp. 6–7).

US schools reopened as vaccinations accelerated. Tully (2021) reported that hybrid learning (i.e., combining remote instruction with in-person classes) marked the transition to reopen school; by early 2021, it was the “most common” arrangement, with thousands of the 13,000 US school districts using it for some or most classes. Tully added, “Whether hybrid classes are helping to stem educational loss remains unclear” (p. 12). The data are sparse, Medina (2021) confirmed, but early reports were worrisome; for example: in November 2020, Austin Beutner, the Los Angeles school superintendent, reported that the district had seen a 15% increase in D’s and F’s among secondary school students, compared with rates the previous year, and a 10% drop in reading proficiency among elementary school students. Will this trend reverse when vaccines are distributed?

While vaccinations have accelerated in wealthy countries, elsewhere the pace has been slow. Dahir and Mueller (2021) reported that across Africa (a continent with 17% of the world’s people), countries had administered only 2% of the vaccine doses given globally; Nigeria is expecting to inoculate only 30% of its people, or about 16 million out of almost 50 million, by 2023. Dahir and Mueller added, “When the rest of the [Nigerian] population will get their shots is anybody’s guess” (A9). Such gross global inequities not only cost lives but livelihoods too: US Federal Reserve chair—Jerome H. Powell—stressed that getting the world vaccinated is critical to the global economic outlook. Speaking to an International Monetary Panel on April 8, 2021, Mr. Powell pointed out that “viruses are no respecters of border”, adding: “Until the world, really, is vaccinated, we’re all going to be at risk of new mutations, and we won’t be able to really resume activity with confidence all around the world” (Smialek 2021, B3). Powell is not alone in emphasizing “how important it is that all nations—not just the richest ones—are able to widely protect against the coronavirus” (Smialek 2021, B3).

International inequities parallel intra-national ones, producing not only public health risks but also educational challenges, evident (for instance) as US schools reopened in early 2021. Shapiro et al. (2021) reported that “hundreds of thousands of Black parents say they are not ready to send their children back” (A1), partly because the virus has sickened and killed non-white Americans disproportionately, but also due to the “profound lack of trust that Black families have in school districts, a longstanding phenomenon exacerbated by the pandemic” (A20). The US Centers for Disease Control and Prevention reported that 82% of White parents strongly or somewhat agreed schools should reopen, while only 46% of Black parents were willing to return their children to classrooms. That difference was not due to frustration with online learning (although there was that), as Harvard’s *Education Next* reported that low-income Black and Latino students were much more likely to be receiving fully remote instruction than higher-income White children. Black parents were 19 percentage points less likely than White parents to prefer in-person learning; Latino parents, 8 percentage points less likely. Shapiro and her colleagues reported that “remote learning has been disastrous for many children of color in particular, and data has shown that students are falling behind in key subjects” (A20). Home schooling among US Black families has been on the rise for years, an effort to protect children from the “hostile school environments”, although not always successfully, as even during home schooling “Black children have continued to be subjected to harsher disciplinary practices, and jarring interactions with school staff” (Shapiro et al. 2021, A20). Predictably, then, large urban school districts, serving tens of thousands of low-income families and, for the most part, operating entirely remotely since March 2020, have reported record-high absentee rates (Goodnough 2021, p. 14).

Asian Americans have also suffered. In March 2021, a gunman killed eight people, including six women of Asian descent, at massage parlors in and near Atlanta, Georgia. Afterward, a poll on Telegram, a messaging app, asked, “Appalled by the recent attacks on Asians?” Alba (2021) reported that the most common answer, with 84% of the vote, was that violence was “justified retaliation for Covid” (A13). Since the outbreak anti-Asian sentiment has “flared up in corners of the internet, amplifying racist and xenophobic tropes”; there have been nearly “eight million mentions of anti-Asian speech online” (A13).

In Canada, teachers have responded to anti-Asian violence by contesting stereotypes in classrooms. Jessica Wong (2021) reported on three teachers’ efforts: Mary Reid, an assistant professor at the Ontario Institute for Studies in Education at the University of Toronto, wrote an impassioned response to the Atlanta murders, stressing the “need to dismantle systemic anti-Asian racism, hand in hand with ongoing efforts against anti-Black and anti-Indigenous racism”. Wong reported as well on Toronto district school board (TDSB) teacher Mary Tran, who discussed the Atlanta mass shooting and anti-Asian racism with her grade-8 virtual school students. Tran is coauthor of the recently released teaching resource: *Addressing Anti-Asian Racism*. Created through a partnership between the TDSB and the Elementary Teachers’ Federation of Ontario, the resource provides historical context as well as questions for reflection, suggesting actions for recognizing and contesting anti-Asian racism within school settings. Finally, Wong reported that Carol Liao, a Vancouver parent of three school-aged children, said she has felt heartened by the efforts of Canadian educators teaching a panoramic picture of Canada’s history and its present. A law professor at the University of British Columbia in Vancouver, to support K-12 educators Liao recently moderated an online discussion of another recently launched free resource called *Challenging Racist “British Columbia”: 150 Years and Counting*. Addressed to teachers, scholars, and policy makers, the book teaches about racism in the province's history and present day.

There is no Covid-19 crisis in China, where the pandemic began but was quickly controlled (Shira and Associates 2021). Two news items remind us that even in a largely Covid-free country, educational issues remain. In China, university graduates face an insufficient number of jobs; many have been rerouted from the workplace to graduate school. The Ministry of Education ordered universities to, however, expand the number of master’s candidates by 189,000, a nearly 25% increase. Undergraduate slots were to be increased by more than 300,000 (Wang 2021, B4). Economic issues are intertwined with ecological ones: on March 15, 2021, an enormous dust storm swept across northern China, grounding hundreds of flights, closing schools, and casting a “ghostly shroud over tens of millions of people”, according to China’s meteorological service (Myers 2021, A10). Following weeks of smog, the storm recalled the “airpocalyses” that China suffered several years ago; in March 2021, Myers reported that,

Three forces—the post-Covid-19 industrial rebound, the continued impact of climate change on the deserts of northern China, and a late winter storm—combined to create a dangerous, suffocating pall. “Beijing is what an ecological crisis looks like”, said Li Shuo, the policy director for Greenspace China, [adding that the] storm was “the result of land and ecological degradation in the north and west of Beijing”. (A10)

Technologists hope to intervene in climate change via “solar geoengineering” (Flavelle 2020, B3). Flavelle also reported that “the idea has been derided as a dangerous and illusory fix, one that would encourage people to keep burning fossil fuels while exposing the planet to unexpected and potential menacing side effects” (B3).

Just as the Covid-19 crisis underscored the centrality of science and technology in humanity’s efforts to survive—vaccinations were produced in record time—(not only) in India is science under assault. There, students are learning that India’s cows have more emotions than foreign cows and that their humps have special powers, part of a new curriculum devised by the National Cow Commission and promoted by Prime Minister Narendra Modi’s Hindu Nationalist government (Gettleman and Raj 2021). In the United States, the lightning-rod issue has not been cows but climate change, indicative of what Brooks terms an “epistemological crisis”, as many Americans are evidently “detached from reality” (Brooks 2021, A24). He continued: “Moreover, this is not just an American problem. All around the world, rising right-wing populist parties are floating on oceans of misinformation and falsehood” (A24).

Educators bear the brunt. Take the case of US science teacher James Sutter, whose lessons on climate change irritated several students, including Gwen Beatty, an Ohio high-school junior. When Mr. Sutter informed students that scientists have linked global warming to heat-trapping gases released by burning fossil fuels, Ms. Beatty interrupted him: “Scientists are wrong all the time”, she asserted, praising then-President Trump’s announcement that the United States would withdraw from the Paris climate accord. Ms. Beatty told him that teachers “are supposed to be open to opinions”. “It’s not about opinions”, Mr. Sutter replied: “It’s about the evidence” (Harmon 2017, A1). “As more of the nation’s teachers seek to integrate climate science in the curriculum”, Harmon reported, “many of them are reckoning with students for whom suspicion of the subject is deeply rooted” (A1).

While many reject technology as an explanation for this “crisis of epistemology”, others point to the “rise of high-hostility, polarizing social media” (Jones 2020, p. 178)—not only for its role in a false “democratization” of knowledge, but also its encouragement of “*violence* … via verbal onslaughts, internet trolling, instantly transmitted and reproduced visual incitements, and all the other virtual means of displaced but no less brutal assaultiveness” (Eley 2020, p. 290). Byung-Chul Han (2017) pointed out:

Initially, the internet was celebrated as a medium of boundless liberty … Today, unbounded freedom and communication are switching over into total control and surveillance. More and more, social media resemble digital panoptical keeping watch over the social realm and exploiting it mercilessly. (p. 8)

Despite these developments—and the disillusion many felt during the pandemic—many remain mired in a techno-utopianism, abetted by technology companies committed to expanding the market for their products. Singer (2017) reported that the big tech companies are taking a “hands-on role in nearly every step of the education supply chain by financing campaigns to alter policy, building learning apps to advance their aims and subsidizing teacher training” (A14); Larry Cuban, an emeritus professor of education at Stanford University, termed this an “almost monopolistic approach to education reform” (A14).

Williamson (2017) reported that Pearson has been trying to “bypass the cumbersome bureaucracy of mass standardized testing and assessment … and instead focus on … the AI-enhanced classroom”, providing “detailed and intimate analytics of individual performance, which will be gained from detailed modelling of learners through their data” (p. 164). In Pearson’s plan, “educational systems” are recast as “neurocomputational networks where brain-based technologies will perform a constant measurement and management of learning environments and of all those individuals who inhabit them” (p. 164). Clearly, technologization continues unabated, admittedly not exactly “breaking news”, as a brief review of its history in the United States reminds us.

## Back to the future

One hundred years ago, it was the radio, not Zoom, that represented “online” learning. The Ohio State School of the Air debuted on January 7, 1929; it was disbanded less than a decade later. The first national school of the air—the RCA Educational Hour—first aired on October 26, 1928; it ceased broadcasting in 1942. The American School of the Air, sponsored by the Columbia Broadcasting System (CBS), began broadcasting on February 4, 1930; it went off the air in 1940. Why? While there was disappointment that radio didn’t result in all children learning what they were taught, the radio also disappeared because it was sidelined by another more “advanced” technological medium, television. “Like the slide, film, radio, and teaching machines”, Saettler (1990) noted that “television as an instructional medium [fell] into general disuse” (p. 388). Will computers, iPads, and other devices follow suit?

The first use of educational films preceded radio remote instruction; it occurred in 1910 in Rochester, New York, the first US public school system to adopt films for regular instructional use. Besides manufacturing cameras, the Rochester-based Eastman Kodak Company created Eastman Teaching Pictures, Inc., producing silent educational films on geography, science, and health; it ceased production in 1944. The next year, Iowa State College (now University) applied to the Federal Communications Commission (FCC) for a construction permit to build an educational television studio; on February 21, 1950, WOI-TV began broadcasting. Licensed by the University of Houston and the Houston Board of Education, KUHT became the first noncommercial station; it began broadcasting on May 12, 1953. After the inauguration of these, others followed; by mid-1955, 16 channels were in operation (Saettler 1990).

Also, in the 1950s—at a conference at the University of Pittsburgh—Skinner (1954) demonstrated a machine that “taught” spelling and arithmetic. Skinner was not the first: Sidney L. Pressey, a psychologist on the faculty of Ohio State University, had exhibited a device anticipating Skinner’s machine at the 1925 meeting of the American Psychological Association (Saettler 1990). Computers became teaching machines, providing, by the 1980s, tutorials, drills, simulations, games, and tests (Alessi and Trollip 1991). Much of the early work on computer-assisted instruction had been done in the 1950s by the International Business Machines Corporation (IBM); by the new millennium, IBM joined with others, including Pearson (noted earlier) and other “ed-tech” companies (Lynch 2020). Also in the 1950s—1956 to be exact—Allan Newell and Herbert A. Simon published a description of a computer program that solved problems by simulating rationality or logic, an early form of artificial intelligence (Saettler 1990, AI).

Problems accompanying the incorporation of computers into the curriculum 40-plus years ago remain today. One was/is the simple availability of computers, including their cost, requiring differential distribution, siphoning off funds from teachers, deepening economic inequality within and across countries. A second problem concerned procedures for purchasing courseware that teachers would not or could not use, triggering a third more general problem: computer literacy (Alessi and Trollip 1991), not so much a problem in wealthy countries today but one that still plagues poorer ones.

Following that first wave of incorporation came critiques, focused at first on many educators’ uncritical enthusiasm for computers and other forms of technology. Sloan (1985) worried that “American educators have made no concerted effort to ask at what level, for what purposes, and in what ways the computer is educationally appropriate and inappropriate, in what ways and to whom we can count on its being beneficial or harmful”, adding: “Professional responsibility demands more” (p. 1). Other critics attended to technologization’s cultural and ecological consequences. “In effect”, Bowers (1988) acknowledged, “a twentieth-century view of knowledge involves using the microcomputer as a powerful and legitimate tool of the teacher and students” (p. 46). But Bowers (1993) pointed out that insofar as computers embody the conceptual framework (and the ideology) of the experts who devise them, technology itself conveys a specific ideological orientation. Bowers also argued that technologization paved over the moral and spiritual character of the ecological crisis, one characterized by the following assumptions: (a) that individual autonomy and rational empowerment are dependent upon data, (b) that change is linear and progressive, (c) a naive anthropocentricity, and (d) a universalization of the assumptions of liberalism, equating these with modernization. Bowers worried about the model of thinking that AI incorporates (i.e., thinking of the new in terms of the familiar) and about the violence of video games. The real crisis of our time, Bowers concluded, is not the lack of data or computer literacy but rather the lack of moral and spiritual development that takes account of the interconnectedness of all life (see Dentith et al. 2022).

Landauer (1988) presciently predicted that “by 2020 a good portion of the cognitive tasks now performed by people will be capable of performance by machine” (p. 19). Stefik (1986) foresaw “an information network with semi-automated services for the generation, distribution, and consumption of knowledge” (pp. 34–35; see also Bereiter and Scardamalia 1992). Goodlad and Su (1992) were clear that:

Curriculum as technology … focuses on process. It is concerned with the technology by which knowledge is communicated and learning is facilitated. Again, it is concerned with the “how” rather than the “what” of education. The function of curriculum is to find efficient means to a set of predefined and, usually, rather simple ends. The focus is on the practical problem of efficiently packaging and presenting the material to the learner, but not on the individuality of the learner or the content. The technologists claim to be developing a value-free system. (p. 335)

Like Bowers (1988, 1993), Aoki (2005) knew that a value-free technology is a contradiction in terms. “I am provoked”, he wrote, “by what I see as the partial blindness of high fashion in the world of Curriculum wherein I see bandied about with almost popular abandon, expressions linked to the computer without a deep understanding of what they are saying”, adding that technologization is “rooted in man’s interest in means, reflects his will to master, to control and to manipulate” (p. 153).

## Conclusion

For many, technology (and critiques of it) are pastimes of the privileged. Even in the United States, where state education and district officials face a myriad of pandemic-intensified problems, including an undersupply of teachers—actual persons not yet replaced by teacher bots (Newton 2018)—causing individual schools and even entire districts to shut down in-person instruction, even for weeks. To address the issue, school districts large and small increased pay for substitute teachers and advertised for temporary positions on local billboards; several states and districts suspended college course requirements and permitted abbreviated online training (Singer 2021). Before the academic year began in August 2020, Los Angeles school district officials distributed hundreds of thousands of laptops and iPads; not only did such purchases reroute funds away from teacher positions and salaries, during the pandemic many teachers discovered to their dismay that “their decades of experience inside classrooms are rendered moot”; students said they missed the “casual helpful interactions that come in classrooms and hallways” (Medina 2021, p. 7). While teachers offered weekend instruction for students who felt lost, “those tutorials can’t totally take the place of in-person instruction” (p. 7). Many students simply disappeared: in the United States, there has been no official accounting of how many students attended school in person or virtually, no record of how many students even bothered to log on; the federal government also failed to track how many Covid-19 cases were identified in schools or which mitigation methods districts are using (Taylor 2021).

While remote learning has proven unsatisfactory for many, few school districts—including, apparently, in Canada—can document the extent of the damage, as few have assessed what students have and have not learned since schools closed in March 2020. Jay Wilson, head of the Department of Curriculum Studies, College of Education, the University of Saskatchewan, acknowledged that “it’s going to take a long time for us to really figure out how it's going to impact [education], but we're seeing all kinds of pressures on students in terms of mental health … all kinds of things that are maybe existing pressures but have been magnified by the pandemic” (Atter 2021, para. 5). A survey undertaken by his institution found that 54% believed “there will be negative long-term impacts on children's education from the pandemic … However, when asked specifically about online learning, 63 per cent [sic] saw it having a positive long-term change” (para. 3).

Apparently, facts do not undermine faith. In the US, however, the data do not invite interpretation. Los Angeles (see above) is not the only city where students have struggled with online learning. In Houston, the United States’ seventh-largest public school district, 42% of students received at least one F in the first grading period in the fall, compared with 26% in the fall of 2019; in the Saint Paul Public Schools (Minnesota), where nearly all students were learning remotely, 32% of grades given in high school core courses were failing marks, up from 12% the year before; in Fairfax County Public Schools in Virginia, where classes were also virtual, the percentage of middle and high school students who failed two or more classes increased 83% from the previous year (Taylor 2021, p. 4). In Baltimore there has been “chaos”, as few “students show up, schedules were rearranged at the last minute, Zoom links were inaccessible”; schools in Hillsborough County, Florida, started the year unable to account for 7,000 missing students; the overall effect of online learning can be summarized as “horrendous” (Brooks 2021, A23).

For students suffering poverty, not learning in person has been accompanied by not eating at all. Taylor (2021) reported that for many of the students who have not set foot in school since March, in-person education also represents a “critical safety net—a source of food and other basic necessities, a place with caring adults who will notice signs of abuse or neglect—from which they are now cut off” (p. 4). There have been psychological consequences as well: Centers for Disease Control and Prevention report that mental health-related visits to hospital emergency rooms by children 5 to 17 years old increased significantly during the pandemic (p. 5). “Although circumstances and solutions across school districts vary widely”, Otis (2021, A2) concluded, “a common reaction became apparent: an intractable worry about the long-term and, as of yet, unknown consequences the disruption of education will have on a generation of children”.

Will the lesson learned during the pandemic—one already taught by the history of technology in US education, sketched above—be forgotten? Despite its disappointments and dangers, will the technologization of education continue unabated, unregulated? In his review of Toby Ord’s *The Precipice: Existential Risk and the Future of Humanity*, Jim Holt (2021) reported that, after reviewing the dangers posed to humanity by nuclear weapons, climate change, and pandemics, Ord deems the most dangerous of all existential threats over the next century to be AI, an assessment shared by figures such as Elon Musk, Bill Gates, Marvin Minsky, and Stephen Hawking. (Holt noted that Mark Zuckerberg disagrees.) That concern is based on predications that AI will not only rival but exceed human intelligence, a prospect that, according to a 2016 survey of 300 top A1 researchers, has a 50-50 chance of occurring within 4 decades, and a 10% chance of occurring in the next 5 years (p. 27).

Despite our utter dependence on—indeed, embeddedness within—technology, is no critique of it worthy of inclusion in the curriculum? Is not Han’s (2017) insight that “technological progress is underpinned by a quasi-religious narrative which assigns it the function of accelerating the arrival of a future salvation” (p. 29) also knowledge of most worth? “We are not robots, machines, or automata”, Rocha (2020) reminds, adding: “We are internally at threat of becoming machines ourselves … The greatest moral danger to the person is their depersonalization … After all, only a person can be educated. Robots are just programmed. Only persons call for leadership. Robots need programmers, not leaders”.

In light of this, must not the student—the individual person—remain central to any conception of curriculum, to any organization of pedagogical communication, indeed to the very project of education itself? I invite you to explore these questions as you read the articles that follow.

## References

[CR1] Alba, D. (2021, March 19). How anti-Asian online posts set the stage for real-world violence. *The New York Times*.https://www.nytimes.com/2021/03/19/technology/how-anti-asian-activity-online-set-the-stage-for-real-world-violence.html

[CR2] Alessi S, Trollip S (1991). Computer-based instruction: Methods and development.

[CR3] Aoki, T. T. (2005). Toward understanding computer application. In W. F. Pinar & R. L. Irwin (Eds.), *Curriculum in a new key: The collected works of Ted T. Aoki* (pp. 151–158). Mahwah, NJ: Lawrence Erlbaum. (Original work published in 1987)

[CR4] Atter, H. (2021, April 4). Head of Curriculum Studies says online learning may lead to positive long-term education changes. *CBC*. https://www.cbc.ca/news/canada/saskatchewan/head-of-curriculum-studies-says-online-learning-may-lead-to-positive-long-term-education-changes-1.5975103

[CR5] Bereiter C, Scardamalia M, Jackson P (1992). Cognition and curriculum. Handbook of research on curriculum.

[CR6] Bosman, J. (2021, January 21). The board voted to keep schools closed. Parents revolted. *The New York Times*. https://www.nytimes.com/2021/01/21/us/coronavirus-schools-wisconsin.html.

[CR7] Bowers CA (1988). Teaching a nineteenth-century mode of thinking through a twentieth-century machine. Educational Theory.

[CR8] Bowers CA (1993). Against the grain: Critical essays on education, modernity, and the recovery of the ecological imperative.

[CR9] Brooks, D. (2021, January 28). Children need to be back in school tomorrow.* The New York Times. *https://www.nytimes.com/2021/01/28/opinion/coronavirus-schools-unions.html.

[CR10] Dahir, A. L., & Mueller, B. (2021, March 23). Some nations could wait years for Covid shots. That’s bad for everyone.* The New York Times.* https://www.nytimes.com/2021/03/22/world/africa/africa-vaccine-inequality-covid.html.

[CR11] Dentith AM, Flinders D, Lupinacci J, Thom JS (2022). Curriculum, environment, and the work of C. A. Bowers: Ecological and cultural perspectives.

[CR12] Dorfman, A. (2020). Songs of loss and reinvention. *The New York Review of Books*, *67*(19), 49–50. https://www.nybooks.com/articles/2020/12/03/songs-of-loss-and-reinvention/.

[CR13] Eley G, Thomas JA, Eley G (2020). Conclusion. Visualizing fascism: The twentieth-century rise of the global right.

[CR15] Gettleman, J., & Raj, S. (2020, September 27). As Covid-19 closes schools, the world’s children go to work.* The New York Times.* https://www.nytimes.com/2020/09/27/world/asia/covid-19-india-children-school-education-labor.html.

[CR16] Gettleman, J., & Raj, S. (2021, February 23). “Weird” course extolling Indian cows stirs ideological fight.* The New York Times*.

[CR17] Goodlad J, Su Z, Jackson P (1992). Organization of the curriculum. Handbook of research on curriculum.

[CR18] Goodnough, A. (2021, January 21). *With students missing online classes, teachers are going to students*. The New York. https://www.nytimes.com/2021/01/21/us/coronavirus-schools-washington-dc.html.

[CR19] Flavelle, C. (2020, November 28). As climate disasters pile up, a radical proposal gains traction. *The New York Times*. https://www.nytimes.com/2020/10/28/climate/climate-change-geoengineering.html?searchResultPosition=1.

[CR20] Han, B.-C. (2017). *The scent of time. A philosophical essay on the art of lingering* (D. Steiner, Trans.). Cambridge, UK: Polity.

[CR21] Harmon, A. (2017, June 4). Climate science meets a stubborn obstacle: Students. *The New York Times*. https://www.nytimes.com/2017/06/04/us/education-climate-change-science-class-students.html.

[CR22] Higgins, A. (2021, February 9). Polish court orders scholars to apologize over Holocaust study. *The New York Times*. https://www.nytimes.com/2021/02/09/world/europe/poland-holocaust-jews-libel.html.

[CR23] Holt, J. (2021). The power of catastrophic thinking. *The New York Review of Books,**68*(3), 26–29. https://www.nybooks.com/articles/2021/02/25/power-catastrophic-thinking-toby-ord-precipice/

[CR24] Jones G (2020). 10% less democracy: Why you should trust elites a little more and the masses a little less.

[CR25] Landauer TK, Zodhiates PP, Nickerson RS (1988). Education in a world of omnipotent and omniscient technology. Technology in education: Looking toward 2020.

[CR26] Lynch, M. (2020, April 21). 10 largest edtech companies. *The Tech Edvocate*. https://www.thetechedvocate.org/10-largest-edtech-companies/.

[CR27] Maclean, R. (2021, March 1). Nigeria’s boarding schools have become a hunting ground for kidnappers. *The New York Times*. https://www.nytimes.com/2021/03/01/world/africa/nigeria-schools-kidnappings.html?searchResultPosition=1.

[CR28] Medina, J. (2021, January 21). In Los Angeles, teachers and students struggle with ‘no human contact’. *The New York Times*. https://www.nytimes.com/2021/01/21/us/coronavirus-schools-los-angeles-california.html.

[CR29] Myers, S. L. (2021, March 15). The worst dust storm in a decade shrouds Beijing and Northern China. *The New York Times*. https://www.nytimes.com/2021/03/15/world/asia/china-sandstorm.html.

[CR51] Newton, D. (2018). Are teachers about to be replaced by bots? *Forbes*. https://www.forbes.com/sites/dereknewton/2018/08/19/are-teachers-about-to-be-replaced-by-bots/?sh=3cbf35dec7c6.

[CR31] Nierenberg, A. (2021, January 25). After the Capitol was stormed, teachers try explaining History in real time.* The New York Times*. https://www.nytimes.com/2021/01/25/us/teaching-capitol-riot.html.

[CR33] Otis, J. (2021, January 22). A slice of what education looks like in pandemic America.* The New York Times*. https://www.nytimes.com/2021/01/22/insider/report-card-story.html.

[CR34] Pinar WF (2019). What is curriculum theory?.

[CR35] Ravitch D (2021). The dark history of school choice. The New York Review of Books.

[CR36] Rocha SD (2020). The syllabus as curriculum: A reconceptualist approach.

[CR37] Saettler P (1990). The evolution of American educational technology.

[CR38] Shapiro, E., Green, E.L., & Kim, J. (2021, February 1). Missing in school reopening plans: Black families’ trust.* The New York Times.* https://www.nytimes.com/2021/02/01/us/politics/school-reopening-black-families.html

[CR39] Shira, D. and Associates (2021, April 9). China coronavirus updates: Latest developments and business advisory.* China Briefing*. https://www.china-briefing.com/news/china-coronavirus-updates-latest-developments-business-advisory-part-2/

[CR40] Singer, N. (2017, June 6). The Silicon Valley billionaires remaking America’s schools. *The New York Times*. https://www.nytimes.com/2017/06/06/technology/tech-billionaires-education-zuckerberg-facebook-hastings.html

[CR41] Singer, N. (2021, January 19). Pandemic teacher shortages imperil in-person schooling.* The New York Times*. https://www.nytimes.com/2021/01/19/us/pandemic-substitute-teacher-shortages.html

[CR42] Skinner BF (1954). The science of learning and the art of reaching. Harvard Educational Review.

[CR43] Sloan D (1985). The computer in education: A critical perspective.

[CR44] Smialek, J. (2021, April 8). As US prospects brighten, Fed’s Powell sees risk in global vaccination pace.* The New York Times*. https://www.nytimes.com/2021/04/08/business/economy/jerome-powell-vaccination-economy.html.

[CR45] Stefik M (1986). The next knowledge medium. AI Magazine.

[CR46] Taylor, K. (2021, January 21). 13,000 school districts, 13,000 approaches to teaching during Covid.* The New York Times*. https://www.nytimes.com/2021/01/21/us/schools-coronavirus.html?searchResultPosition=1

[CR47] Tully, T. (2021, January 21). ‘The word of the year is fluid’: The pandemic brings a new teaching style.* The New York Times*. https://www.nytimes.com/2021/01/21/us/schools-coronavirus.html?searchResultPosition=1

[CR48] Wang, V. (2021, January 18). China’s college graduates can’t find jobs. The solution: Grad school.* The New York Times*. https://www.nytimes.com/2021/01/18/business/china-graduate-school-white-collar.html?searchResultPosition=1

[CR49] Williamson B (2017). Big data in education: The digital future of learning, policy and practice.

[CR50] Wong, J. (2021, March 30). How teachers are fighting damaging stereotypes in class amid spike in anti-Asian attacks*. CBS News.* https://www.cbc.ca/news/canada/education-anti-asian-racism-1.5968251

